# Supplementary material for: Splice-Junction-Based Mapping of Alternative Isoforms in the Human Proteome
Source: Cell Rep. Author manuscript; Available in PMC 2020 Jan 15. (PMC6961840; doi:10.1016/j.celrep.2019.11.026)
Supplement: 2 [file NIHMS1546469-supplement-2.zip › DF1/PXD010154/Spleen/Spleen_15_RBM39_QILLLFKVYMLEAPYK.pdf]

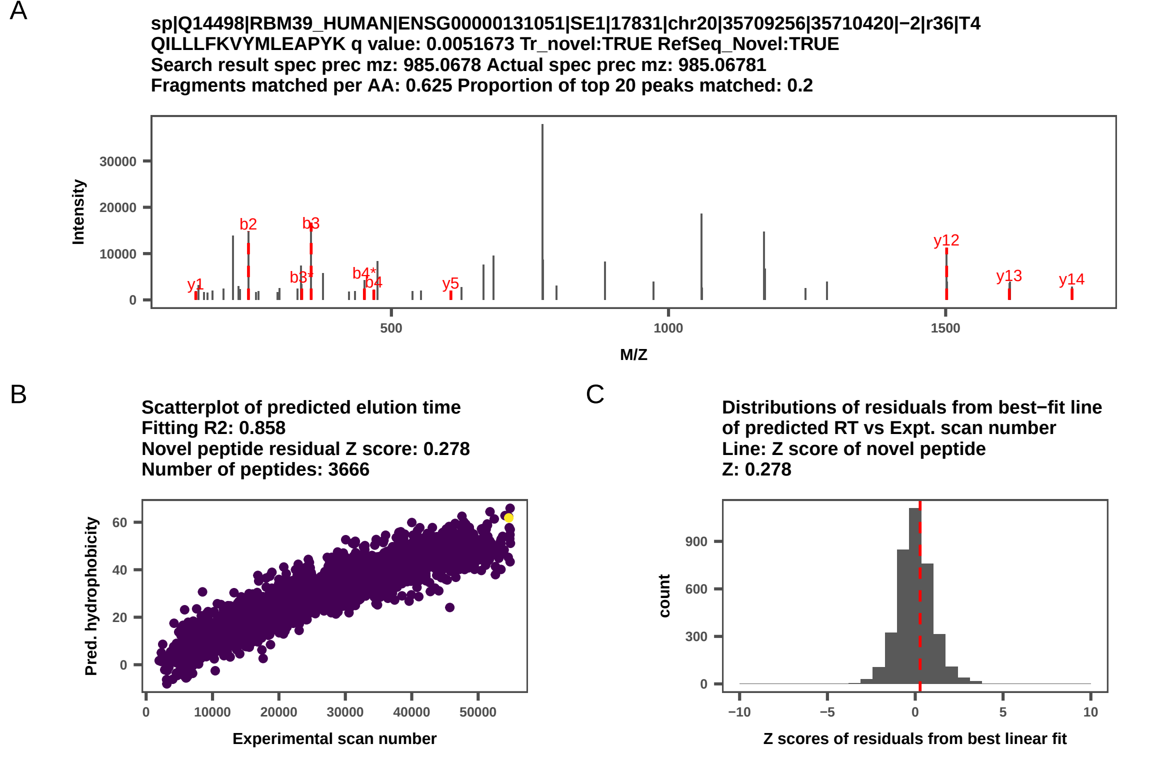
A
sp|Q14498|RBM39_HUMAN|ENSG00000131051|SE1|17831|chr20|35709256|35710420|−2|r36|T4
QILLLFKVYMLEAPYK q value: 0.0051673 Tr_novel:TRUE RefSeq_Novel:TRUE
Search result spec prec mz: 985.0678 Actual spec prec mz: 985.06781
Fragments matched per AA: 0.625 Proportion of top 20 peaks matched: 0.2
Intensity
30000
20000
10000
0
y1
b2
b3*
b3
b4*
b4
y5
y12
y13
y14
500
1000
1500
M/Z
B
Scatterplot of predicted elution time
Fitting R2: 0.858
Novel peptide residual Z score: 0.278
Number of peptides: 3666
Pred. hydrophobicity
60
40
20
0
0
10000
20000
30000
40000
50000
Experimental scan number
C
Distributions of residuals from best−fit line
of predicted RT vs Expt. scan number
Line: Z score of novel peptide
Z: 0.278
count
900
600
300
0
−10
−5
0
5
10
Z scores of residuals from best linear fit
